# Expression of HGF, pMet, and pAkt is related to benefit of radiotherapy after breast‐conserving surgery: a long‐term follow‐up of the SweBCG91‐RT randomised trial

**DOI:** 10.1002/1878-0261.12803

**Published:** 2020-09-28

**Authors:** Martin Sjöström, Cynthia Veenstra, Erik Holmberg, Per Karlsson, Fredrika Killander, Per Malmström, Emma Niméus, Mårten Fernö, Olle Stål

**Affiliations:** ^1^ Division of Oncology and Pathology Department of Clinical Sciences Lund Faculty of Medicine Lund University Lund Sweden; ^2^ Department of Biomedical and Clinical Sciences Linköping University Linköping Sweden; ^3^ Department of Oncology Linköping University Linköping Sweden; ^4^ Department of Oncology Institute of Clinical Sciences Sahlgrenska Academy Sahlgrenska University Hospital University of Gothenburg Gothenburg Sweden; ^5^ Department of Haematology, Oncology and Radiation Physics Skåne University Hospital Lund Sweden; ^6^ Division of Surgery Department of Clinical Sciences Lund Faculty of Medicine Lund University Lund Sweden; ^7^ Department of Surgery Skåne University Hospital Lund Sweden

**Keywords:** Akt, breast cancer, HGF, Met, radiotherapy, treatment prediction

## Abstract

Experimental studies suggest that hepatocyte growth factor (HGF) and its transmembrane tyrosine kinase receptor, Met, in part also relying on Akt kinase activity, mediate radioresistance. We investigated the importance of these biomarkers for the risk of ipsilateral breast tumour recurrence (IBTR) after adjuvant radiotherapy (RT) in primary breast cancer. HGF, phosphorylated Met (pMet) and phosphorylated Akt (pAkt) were evaluated immunohistochemically on tissue microarrays from 1004 patients in the SweBCG91‐RT trial, which randomly assigned patients to breast‐conserving therapy, with or without adjuvant RT. HGF was evaluated in the stroma (HGF_str_); pMet in the membrane (pMet_mem_); HGF, pMet and pAkt in the cytoplasm (HGF_cyt_, pMet_cyt_, pAkt_cyt_); and pAkt in the nucleus (pAkt_nuc_). The prognostic and treatment predictive effects were evaluated to primary endpoint IBTR as first event during the first 5 years. Patients with tumours expressing low levels of HGF_cyt_ and pMet_cyt_ and high levels of pAkt_nuc_ derived a larger benefit from RT [hazard ratio (HR): 0.11 (0.037–0.30), 0.066 (0.016–0.28) and 0.094 (0.028–0.31), respectively] compared to patients with high expression of HGF_cyt_ and pMet_cyt_, and low pAkt_nuc_ [HR: 0.36 (0.19–0.67), 0.35 (0.20–0.64) and 0.47 (0.32–0.71), respectively; interaction analyses: *P* = 0.052, 0.035 and 0.013, respectively]. These differences remained in multivariable analysis when adjusting for patient age, tumour size, histological grade, St Gallen subtype and systemic treatment (interaction analysis, *P*‐values: 0.085, 0.027, and 0.023, respectively). This study suggests that patients with immunohistochemically low HGF_cyt_, low pMet_cyt_ and high pAkt_nuc_ may derive an increased benefit from RT after breast‐conserving surgery concerning the risk of developing IBTR.

## Graphical abstract

We investigated the importance of HGF, pMET and pAkt for the risk of ipsilateral breast tumour recurrence after adjuvant radiotherapy in primary breast cancer in tumours from the SweBCG91‐RT randomised trial. Low levels of HGF and pMet in the tumour cell cytoplasm and high levels of pAkt in the tumour cell nucleus are associated with a larger benefit from RT, indicating that these proteins may be used as biomarkers or treatment targets.

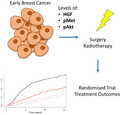

**Abbreviations**
CI: confidence interval
cyt: cytoplasm
ER: oestrogen receptor alpha
FU: follow‐up
HER2: human epidermal growth factor receptor 2
HGF: hepatocyte growth factor
HR: hazard ratio
IBTR: ipsilateral breast tumour recurrence
mem: plasma membrane
nuc: nucleus
pAkt: phosphorylated Akt
pMet: phosphorylated Met
PR: progesterone receptor
RT: radiotherapy
SweBCG91RT: Swedish breast cancer group 91 radiotherapy

## 1 Introduction

Most women with a breast cancer diagnosis are treated with breast‐conserving surgery followed by adjuvant whole‐breast radiotherapy (RT). RT after breast‐conserving surgery approximately halves the risk of ipsilateral breast tumour recurrence (IBTR) [1]. Although the absolute risk reduction varies according to prognostic factors, such as age, tumour size and histological grade, the relative benefit is about the same [1]. There is a need for new biomarkers that either could improve the identification of patients who could be spared RT or to identify those who would need intensified treatment. As reviewed by Forker *et al*. [2], there are several candidate biomarkers concerning radiosensitivity, but further validation is needed from randomised clinical trials.

Amongst growth factor receptors related to apoptosis and DNA repair, that in turn affect radiosensitivity, is the hepatocyte growth factor (HGF) receptor Met. Experimental studies have shown that ionising radiation induces increased expression and activation of Met [3]. Moreover, Met inhibition suppressed radioresistance *in vitro* [4, 5], and elevated tumour levels of HGF and Met were associated with a worse prognosis for rectal cancer patients treated with chemo‐radiotherapy [5]. The protein kinase Akt, activated downstream of Met and other growth factor receptors, is another protein that has been linked to radioresistance in experimental studies [6, 7]. Both Met and Akt can be targeted, and inhibitors are in clinical use or being tested in clinical trials of advanced cancer. Given the experimental data, these inhibitors might also have the potential to increase the effect of RT.

The SweBCG91‐RT trial included patients with lymph node‐negative, stage I and IIA breast cancer, randomly assigned to breast‐conserving surgery, with or without whole‐breast RT [8]. Based on clinically used breast cancer markers, all subgroups of patients benefited from RT [9], with some uncertainty regarding human epidermal growth factor receptor 2 (HER2)‐positive disease [10]. However, a recently reported clinico‐genomic classifier was validated to be both prognostic and predictive for RT in this breast cancer cohort [11]. In the present study, the aim was to assess whether the expression of HGF, phosphorylated Met (pMet) and activated Akt (pAkt) predicts radiosensitivity in a large randomised trial of patients treated with breast‐conserving surgery with or without RT and largely systemically untreated. We hypothesise that overexpression of these markers predicts decreased radiosensitivity. There might, however, be functional differences related to the subcellular localisation of the proteins. Studies by Oeck *et al*. [12] suggest that radiosensitivity might depend on the cellular localisation of pAkt and the associations of nuclear and cytoplasmic pAkt with breast cancer subtype differ [13]. Likewise, the relative distribution of membraneous and cytoplasmic Met was found to be important for the prognosis of colon cancer [14]. Therefore, we chose to evaluate the nuclear and cytoplasmic expression of pAkt and the cytoplasmic and membraneous expression of Met separately.

This unique cohort, with approximately half of the patients treated without postoperative therapy and with minimal use of systemic adjuvant therapy, allows for analysis of the prognostic and predictive value concerning radiotherapy separately and without confounding from other treatments.

## 2 Materials and methods

### 2.1 Patients and study design

Patients with lymph node‐negative, stage I and IIA breast cancer from the SweBCG91‐RT trial were included. Between 1991 and 1997, patients received breast‐conserving surgery and were randomly assigned between whole‐breast RT or no RT, as previously described [8, 9]. According to regional guidelines at that time, only 6% received endocrine treatment, 1% chemotherapy and 1% endocrine treatment plus chemotherapy. Paraffin‐embedded tissue of the primary tumour could be retrieved from 1004 of the original 1178 patients. This material was used for the re‐evaluation of histological grade according to Elston and Ellis [15] and for the construction of tissue microarrays (TMAs). Oestrogen receptor alpha (ER), progesterone receptor (PR), HER2 and Ki67 were analysed on 1.0 mm cores, as previously described [10]. After that, the tumours were subtyped according to the St Gallen surrogate definition of the intrinsic subtypes 2013 as luminal A‐like (ER‐positive, PR‐positive, HER2‐negative and Ki67 low), luminal B‐like [ER‐positive, PR low (< 20%) and/or Ki67 high, and HER2‐negative], HER2‐positive (HER2‐positive, any ER and PR status and any Ki67) and triple‐negative (ER‐negative, PR‐negative, HER2‐negative and any Ki67 [10].

The ethical committee approved the trial and follow‐up studies, and this study was conducted in accordance with the declaration of Helsinki. The REMARK guidelines for reporting of tumour biomarker studies were followed [16].

### 2.2 Immunohistochemical analysis of pMet, HGF, and pAkt

Tissue microarrays were sliced into 3–4 µm sections and transferred to frost‐coated microscope slides. The sections were covered in a protective layer of paraffin and stored at 4 °C. The paraffin layers were cleared from the slides by upright incubation at 60 °C for 2 h prior analysis. Pretreatment of the TMAs (deparaffinisation, rehydration and epitope retrieval) was performed in the PT Link (Agilent Dako, Santa Clara, CA, USA) with DAKO PT Buffer [Envision FLEX target retrieval solution low (HGF, pAkt) or high (pMet), Agilent Dako]. TMA sections were then incubated with 3% H_2_O_2_ solution to minimise nonspecific staining, followed by serum‐free protein block for 15 min to reduce unspecific binding (Spring Bioscience, Fremont, CA, USA). Sections were incubated overnight at 4 °C with primary antibodies diluted in DAKO Ab diluent (Agilent Dako) against pMet‐Tyr1349 (ab68141, 1 : 50; Abcam, Cambridge, UK), HGF (LS‐B3265, 1 : 200; LifeSpan Bio Sciences Inc., Seattle, WA, USA) and pAkt‐Ser473 (#4060, 1 : 10; Cell Signaling Technology, Beverly, MA, USA). The secondary antibody (HistoPlus HRP One‐Step polymer anti‐Mouse/Rabbit/Rat; Nordic Biosite, Täby, Sweden) was applied for 30 min at room temperature. Colour was developed with liquid DAB+ (Agilent Dako) followed by counterstaining with Mayer's haematoxylin (Merck Sigma‐Aldrich, Darmstadt, Germany). The tissue sections were dehydrated using a series of ethanol dilutions. Whole‐slide images were obtained with Aperio ScanScope AT (Leica Microsystems, Wetzlar, Germany).

**Fig. 1:**
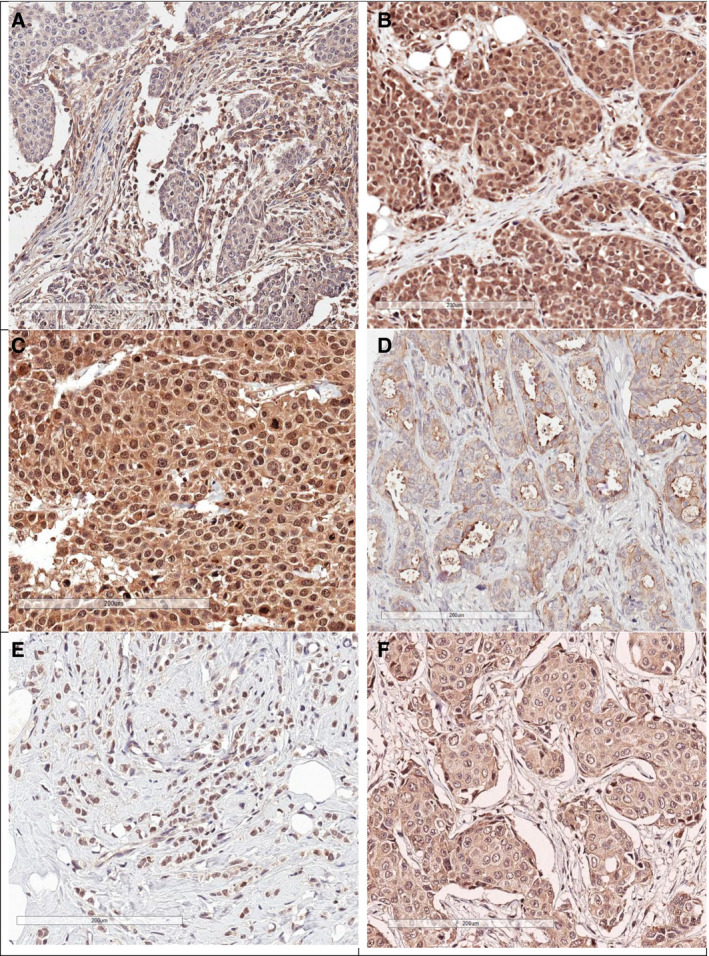
Tumour samples with immunohistological staining of HGF (A, B), pMet (C, D) and pAkt (E, F), representing stromal and low cytoplasmic expression (A), low stromal and high cytoplasmic expression (B), high cytoplasmic expression (C), membrane expression with low cytoplasmic expression (D), nuclear expression and low cytoplasmic expression (E) and high cytoplasmic without nuclear expression (F). Bar is 200 µm.

The immunostaining was graded by two independent researchers (CV and OS) without knowledge of the clinical data per previously obtained guidelines [17]. In short, the membrane scoring of pMet (pMet_mem_) was either negative or positive, as was stromal staining of HGF (HGF_str_). Cytoplasmic staining for HGF (HGF_cyt_) was divided into low (negative or moderate staining) and high (strong staining). pAkt (pAkt_cyt_) and pMet (pMet_cyt_) expression in the cytoplasm was scored as low (negative to weak staining) or high (moderate or strong staining). Nuclear pAkt (pAkt_nuc_) was scored as low (≤ 10% stained nuclei, independent of intensity, or > 10% stained nuclei with a low staining intensity) or high (> 10% strongly stained nuclei). In the case of discordant scoring, the sample was re‐examined, and a joint score was made. Titration experiments were performed to assess the optimal antibody concentration that gave the best staining with minimum background. The antibodies were used and validated in previous studies [17], and representative images for HGF, pMet and pAkt are shown in Fig. 1.

### 2.3 Statistical methods

All statistical analyses were performed in r version 3.6.2 [18]. Primary endpoint was cumulative incidence of IBTR as first or synchronous event, considering regional and distant metastasis and death as competing events. Secondary endpoint was any breast cancer recurrence (local, regional or distant metastasis, but not contralateral breast cancer), considering death without recurrence as competing event. Median follow‐up was 15.2 years for patients free from event. Cumulative incidences were calculated and visualised using the cmprsk v.2.2‐9 package [19]. To contrast hazard rates differences, hazard ratios (HRs) were calculated with cause‐specific Cox regression modelling using the survival v.2.38 package [20]. Since HRs for this study have been shown to be nonproportional over the entire follow‐up time [11, 21], we provide HR estimates for the full follow‐up time and for periods 0–5 years and 5–15 years, and the HRs should be interpreted as the mean over the period studied. Interaction tests were performed for the first 5 years of follow‐up for endpoint IBTR and for full follow‐up for endpoint any recurrence, as we have previously shown that RT has the largest effect on IBTR for the first 5 years, while other recurrences might take longer time to develop [9]. No IBTRs occurred during the first 5 years in the RT‐treated and pAkt_nuc_ high group, and the calculation of HRs and interaction for pAkt_nuc_ were therefore made for the first 10 years. Forest plots were created using the forest plot v.1.9 package [22]. No strict cut‐off of statistical significance was used, but *P*‐values around and below 0.05 were regarded as showing moderate evidence against the null‐hypothesis, and *P*‐values below 0.001 were regarded as strong evidence against the null‐hypothesis.

## 3 Results

### 3.1 Expression of MET, HGF and Akt and association with clinical variables

**Fig. 2:**
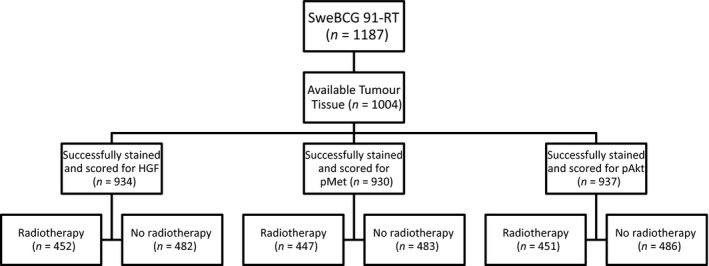
CONSORT diagram showing patients from the SweBCG91‐RT study included in the current study using tissue microarrays.

We were able to score more than 90% of the 1004 retrieved tumours (Fig. 2). The tumours included in the TMA have previously been shown to be representative of the full study, except for including fewer of the smallest tumours [10]. High HGF_str_ and high HGF_cyt_ were found in 45% (416/934) and 66% (615/934), respectively, of the evaluable tumours (Table 1). The corresponding numbers for high pMet_mem_ and high pMet_cyt_ were 31% (287/930) and 66% (616/930) and for high pAkt_cyt_ and high pAkt_nuc_ 48% (449/937) and 26% (243/937) (Tables 1 and 2).

**Table 1:** Patient and tumour characteristics in association to HGF_str_, HGF_cyt_, pMet_mem_ and pMet_cyt_.

*n* (%)	All	HGF_str_		HGF_cyt_		pMet_cyt_		pMet_mem_	
		Neg	Pos	Low	High	Low	High	Low	High
Total	1004	518 (55)	416 (45)	319 (34)	615 (66)	314 (34)	616 (66)	643 (69)	287 (31)
Premenopausal	200 (20)	104 (54)	87 (46)	72 (38)	119 (62)	63 (33)	129 (67)	127 (66)	65 (34)
Postmenopausal	779 (80)	402 (56)	318 (44)	234 (32)	486 (68)	243 (34)	472 (66)	498 (70)	217 (30)
Missing	25	12	11	13	10	8	15	18	5
Tumour size									
1–10 mm	390 (39)	195 (55)	160 (45)	116 (33)	239 (67)	114 (32)	240 (68)	242 (68)	112 (32)
11–20 mm	523 (52)	274 (56)	217 (44)	172 (35)	319 (65)	165 (34)	326 (66)	346 (70)	145 (30)
> 20 mm	85 (9)	45 (55)	37 (45)	29 (35)	53 (65)	32 (41)	47 (59)	52 (66)	27 (34)
Missing	6	4	2	2	4	3	3	3	3
Histological grade									
1	148 (15)	81 (58)	58 (42)^$^	49 (35)	90 (65)	47 (34)	91 (66)	110 (80)	28 (20)^#^
2	573 (60)	313 (59)	216 (41)	188 (36)	341 (64)	187 (35)	343 (65)	366 (69)	164 (31)
3	237 (25)	98 (42)	134 (58)	72 (31)	160 (69)	73 (32)	156 (68)	146 (64)	83 (36)
Missing	46	26	8	10	24	7	26	21	12
ER status									
Negative	101 (10)	36 (37)	61 (63)^$^	31 (32)	66 (68)	29 (31)	65 (69)	60 (64)	34 (36)
Positive	863 (90)	466 (57)	348 (43)	281 (35)	533 (65)	278 (34)	536 (66)	569 (70)	245 (30)
Missing	40	16	7	7	16	7	15	14	8
PR status									
Negative	200 (21)	94 (49)	96 (51)	64 (34)	126 (66)	54 (29)	131 (71)	121 (65)	64 (35)
PR positive	764 (79)	408 (57)	313 (43)	248 (34)	473 (66)	253 (35)	470 (65)	508 (70)	215 (30)
Missing	40	16	7	7	16	7	15	14	8
HER2 status									
Negative	895 (93)	472 (56)	371 (44)	295 (35)	548 (65)	292 (35)	549 (65)*	601 (71)	240 (29)^$^
Positive	64 (7)	28 (44)	35 (56)	16 (25)	47 (75)	13 (21)	49 (79)	24 (39)	38 (61)
Missing	45	18	10	8	20	9	18	18	9
Ki67 status									
Low	719 (75)	391 (58)	278 (42)^$^	228 (34)	441 (66)	242 (36)	425 (64)^#^	492 (74)	175 (26)^$^
High	245 (25)	111 (46)	131 (54)	84 (35)	158 (65)	65 (27)	176 (73)	137 (57)	104 (43)
Missing	40	16	7	7	16	7	15	14	8
Subtype									
Luminal A‐like	555 (58)	307 (59)	209 (41)^#^	175 (34)	341 (66)	189 (36)	331 (64)	391 (75)	129 (25)^$^
Luminal B‐like	259 (27)	134 (54)	116 (46)	93 (37)	157 (63)	76 (31)	170 (69)	155 (63)	91 (37)
HER2+	64 (7)	28 (44)	35 (56)	16 (25)	47 (75)	13 (21)	49 (79)	24 (39)	38 (61)
Triple negative	81 (8)	31 (40)	46 (60)	27 (35)	50 (65)	27 (36)	48 (64)	55 (73)	20 (27)
Missing	45	18	10	8	20	9	18	18	9
HGF_str_									
Negative	518 (55)			245 (47)	273 (53)^$^	192 (38)	315 (62)^#^	343 (68)	164 (32)
Positive	416 (45)			74 (18)	342 (82)	113 (28)	296 (72)	286 (70)	123 (30)
Missing	70			0	0	9	5	14	0
HGF_cyt_									
Low	319 (34)					138 (44)	173 (56)^$^	211 (68)	100 (32)
High	615 (66)					167 (28)	438 (72)	418 (69)	187 (31)
Missing	70					9	5	14	0
pMet_cyt_									
Low	314 (34)							274 (87)	40 (13)^$^
High	616 (66)							369 (60)	247 (40)
Missing	74							0	0

* *P* = 0.049–0.01.

^#^ *P* = 0.009–0.001.

^$^ *P* < 0.001.

**Table 2:** Patient and tumour characteristics in association to pAkt_cyt_ and pAkt_nuc_.

*n* (%)	All	pAkt_cyt_		pAkt_nuc_	
		Low	High	Low	High
Total	1004	488 (52)	449 (48)	694 (74)	243 (26)
Premenopausal	200 (20)	99 (52)	92 (48)	147 (77)	44 (23)
Postmenopausal	779 (80)	378 (52)	345 (48)	531 (73)	192 (27)
Missing	25	11	12	16	7
Tumour size					
1–10 mm	390 (39)	194 (54)	162 (46)	251 (71)	105 (29)
11–20 mm	523 (52)	245 (50)	248 (50)	373 (76)	120 (24)
> 20 mm	85 (9)	47 (57)	35 (43)	64 (78)	18 (22)
Missing	6	2	4	6	0
Histological grade					
1	148 (15)	85 (62)	53 (38)^$^	87 (63)	51 (37)^$^
2	573 (60)	296 (56)	237 (44)	380 (71)	153 (29)
3	237 (25)	92 (40)	139 (60)	207 (90)	24 (10)
Missing	46	15	20	20	15
ER status					
Negative	101 (10)	24 (25)	72 (75)^$^	85 (89)	11 (11)^$^
Positive	863 (90)	456 (56)	363 (44)	596 (73)	223 (27)
Missing	40	8	14	13	9
PR status					
Negative	200 (21)	73 (39)	114 (61)^$^	155 (83)	32 (17)^#^
PR positive	764 (79)	407 (56)	321 (44)	526 (72)	202 (28)
Missing	40	8	14	13	9
HER2 status					
Negative	895 (93)	451 (53)	396 (47)	623 (74)	224 (26)
Positive	64 (7)	26 (41)	37 (59)	53 (84)	10 (16)
Missing	45	11	16	18	9
Ki67 status					
Low	719 (75)	392 (58)	283 (42)^$^	485 (72)	190 (28)^#^
High	245 (25)	88 (37)	152 (63)	196 (82)	44 (18)
Missing	40	8	14	13	9
Subtype					
Luminal A‐like	555 (58)	304 (58)	220 (42)^$^	371 (71)	153 (29)^#^
Luminal B‐like	259 (27)	129 (52)	118 (48)	185 (75)	62 (25)
HER2+	64 (7)	26 (41)	37 (59)	53 (84)	10 (16)
Triple negative	81 (8)	18 (24)	58 (76)	67 (88)	9 (12)
Missing	45	11	16	18	9
HGF_str_					
Negative	518 (55)	291 (57)	219 (43)^$^	382 (75)	128 (25)
Positive	416 (45)	187 (45)	224 (55)	302 (73)	109 (27)
Missing	70	10	6	10	6
HGF_cyt_					
Low	319 (34)	214 (68)	99 (32)^$^	227 (73)	86 (27)
High	615 (66)	264 (43)	344 (57)	457 (75)	151 (25)
Missing	70	10	6	10	6
pMet_cyt_					
Low	314 (34)	202 (66)	105 (34)^$^	247 (80)	60 (20)^#^
High	616 (66)	271 (44)	338 (56)	438 (72)	171 (28)
Missing	74	15	6	9	12
pMet_mem_					
Negative	643 (69)	353 (56)	278 (44)^$^	508 (81)	123 (19)^$^
Positive	287 (31)	120 (42)	165 (58)	177 (62)	108 (38)
Missing	74	15	6	9	12
pAkt_cyt_					
Low	488 (52)			383 (78)	105 (22)^#^
High	449 (48)			311 (69)	138 (31)
Missing	67			0	0

*P* = 0.049–0.01.

a *P* = 0.009–0.001.

b *P* < 0.001.

High HGF_str_ was associated with aggressive tumour characteristics (higher histological grade, ER negativity, high Ki67), whereas HGF_cyt_ showed no marked association with established prognostic factors (Table 1). Like high HGF_str_, high pMet_mem_, high pMet_cyt_ and high pAkt_cyt_ were also associated with more aggressive tumour characteristics, whereas high pAkt_nuc_ was associated with ER and PR positivity, low Ki67 and lower histological grade (Tables 1 and 2). Moreover, pMet_mem_ was strongly positively associated with HER2 status. The experimental biomarkers were, for most combinations, positively associated with one another (Tables 1 and 2).

### 3.2 The treatment predictive value of HGF, pMet and pAkt for radiotherapy

#### 3.2.1 Benefit from radiotherapy for ipsilateral breast tumour recurrence depending on expression of HGF, pMet and pAkt

**Fig. 3:**
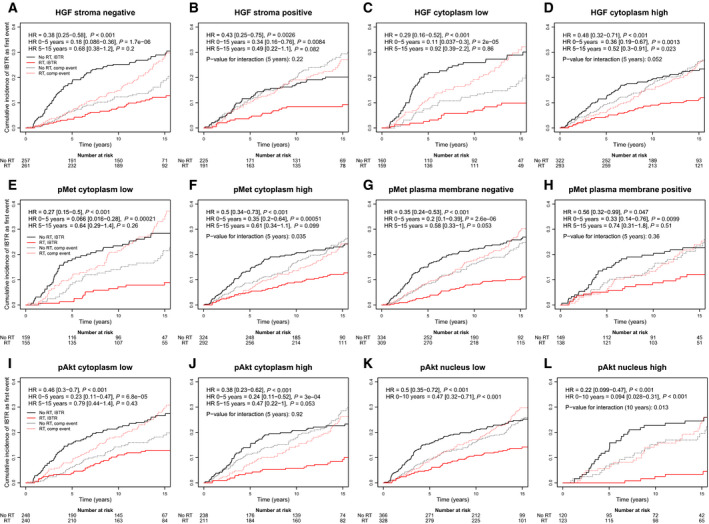
Effect of adjuvant whole‐breast RT in the SweBCG91‐RT study on the cumulative incidence of IBTR for different levels of HGF_str_ (A, B), HGF_cyt_ (C, D), pMet_cy_ (E, F), pMet_mem_ (G, H), pAkt_cyt_ (I, J) and pAkt_nuc_ (K, L). Solid lines represent the cumulative incidence of IBTR, while dashed lines represent the cumulative incidence of competing events.

In the RT‐treated group, the rate of IBTR was 56/485 at full follow‐up time and 19/485 at 5 years, while the rate in the no RT group was 122/519 at full follow‐up and 76/519 at 5 years. Patients with breast cancers with low HGF_cyt_, low pMet_cyt_ and high pAkt_nuc_ derived a larger benefit from RT compared to patients with high HGF_cyt_, high pMet_cyt_ and low pAkt_nuc_ tumours (Fig. 3): HGF_cyt_ (low *vs*. high; 5 years follow‐up): HR = 0.11, 95% confidence interval (CI): 0.037–0.30 *vs*. HR = 0.36, 95% CI: 0.19–0.67 (interaction analysis, *P* = 0.052), pMet_cyt_ (low *vs*. high; 5 years follow‐up): HR = 0.066, 95% CI: 0.16–0.28 *vs*. HR = 0.35, 95% CI: 0.20–0.64 (interaction analysis, *P* = 0.035) and pAkt_nuc_ (high *vs*. low; 10 years of follow‐up): 0.094 95% CI: 0.028–0.31 *vs*. 0.47 95% CI: 0.32–0.71 (interaction analysis, *P* = 0.013). The interaction between RT and HGF_cyt_, pMet_cyt_ and pAkt_nuc,_ respectively, remained in multivariable analyses when adjusting for patient age, tumour size, histological grade, St Gallen subtype and systemic treatment (interaction analysis, *P*‐values: 0.085, 0.027 and 0.023, respectively).

The evidence for an interaction between RT and the expression of these biomarkers became weaker when considering the full follow‐up time (univariable analysis: *P* = 0.16, 0.10, and 0.066, respectively).

#### 3.2.2 Benefit from radiotherapy for any breast cancer recurrence depending on expression of HGF, pMet and pAkt

**Fig. 4:**
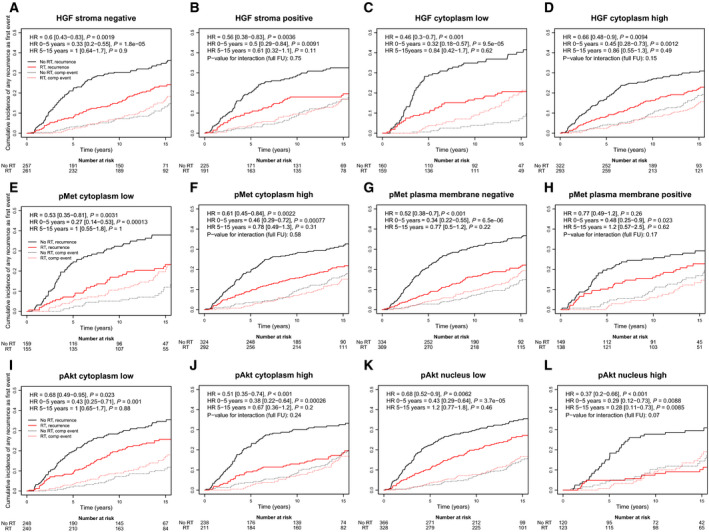
Effect of adjuvant whole‐breast RT on the cumulative incidence of any recurrence for different levels of HGF_str_ (A, B), HGF_cyt_ (C, D), pMet_cyt_ (E, F), pMet_mem_ (G, H), pAkt_cyt_ (I, J) and pAkt_nuc_ (K, L). Solid lines represent the cumulative incidence of any breast cancer recurrence, while dashed lines represent the cumulative incidence of competing events.

A benefit of RT for endpoint any recurrence was found in the full cohort included in the TMA; in the RT‐treated group, the rate of any recurrence was 106/485 at full follow‐up, while the rate in the no RT arm was 169/519 at full follow‐up. In agreement with the findings for IBTR alone, the effect of RT was more pronounced for patients with breast cancer with low HGF_cyt_ or high pAkt_nuc_ (*P*‐values for the interactions of 0.15 and 0.070, respectively; Fig. 4, full follow‐up). A tendency for an increased benefit of RT was also found for patients with low pMet_mem_ tumours compared to patients with high pMet_mem_ tumours (interaction analysis: *P* = 0.17). These interactions were similar in multivariable analyses when adjusting for patient age, tumour size, histological grade, St Gallen subtype and systemic treatment (interaction analysis (whole follow‐up), *P*‐values: 0.12, 0.16 and 0.18, respectively (Fig. 4).

### 3.3 The prognostic value of HGF, pMet and pAkt

#### 3.3.1 Prognosis of Ipsilateral breast tumour recurrence depending on the expression of HGF, pMet and pAkt

**Fig. 5:**
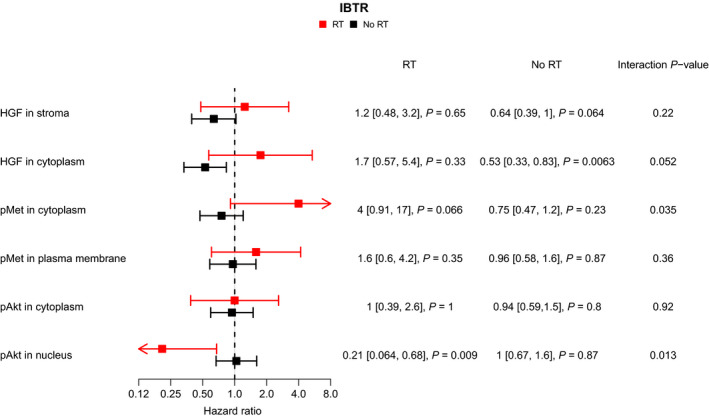
Hazard ratios for development of IBTR within 5 years based on high/positive *vs*. low/negative HGF_str_ HGF_cyt_, pMet_cyt_, pMet_mem_, pAkt_cyt_ and pAkt_nuc_ scoring in the SweBCG91‐RT study, for patients treated with or without RT. The calculation of hazard ratios and interaction for pAkt_nuc_ was made for 10 years of follow‐up. *P*‐values for the respective variables or interaction term were calculated from the Cox regression model using the Wald test.

After 5 years of follow‐up in the group without RT, the incidence of IBTR was in univariable analysis lower for patients with HGF_cyt_ high compared to patients with HGF_cyt_ low tumours (HR = 0.53, 95% CI: 0.33–0.83, *P* = 0.0063; Fig. 5). A similar result was obtained in multivariable analysis, adjusting for patient age, tumour size, histological grade, St Gallen subtype and systemic treatment (HR = 0.57, 95% CI: 0.34–0.94, *P* = 0.027). In the RT‐treated group, patients with high pAkt_nuc_ tumours had a lower incidence of IBTR compared to patients with low pAkt_nuc_ tumours (10‐year follow‐up; HR = 0.21, 95% CI: 0.064–0.68, *P* = 0.009), which remained in the multivariable analysis (10‐year follow‐up; HR = 0.21, 95% CI: 0.063–0.68, *P* = 0.009). For the remaining experimental biomarkers, no differences after 5 years of follow‐up were found in univariable analysis between high *vs*. low content in neither the group without RT nor the group with RT (Fig. 5 and Fig. S1).

#### 3.3.2 Prognosis of any recurrence depending on the expression of HGF, pMet and pAkt

**Fig. 6:**
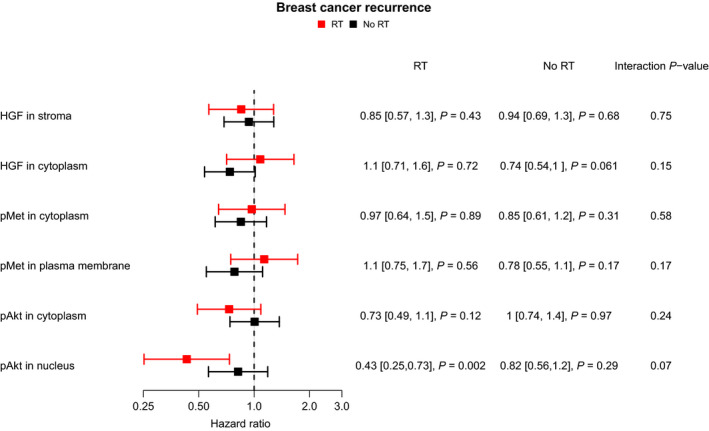
Hazard ratios for development of any recurrence during the full follow‐up based on high/positive *vs*. low/negative HGF_str_, HGF_cyt_, pMet_cyt_, pMet_mem_, pAkt_cyt_ and pAkt_nuc_ scoring in the SweBCG91‐RT study, for patients treated with or without RT. *P*‐values for the respective variables or interaction term were calculated from the Cox regression model using the Wald test.

When using any recurrence during full follow‐up as endpoint, a similar pattern was found (Fig. 6 and Fig. S2). There was moderate support by statistical testing for the difference between low and high HGF_cyt_ in the no RT group (univariable analysis: HR = 0.74, 95% CI: 0.54–1.0, *P* = 0.061; multivariable analysis: HR = 0.72, 95% CI = 0.51–1.0, *P* = 0.058). For pAkt_nuc_ (high *vs*. low) in the RT‐treated group, there was a prognostic difference in univariable analysis for the rate of any recurrence (HR = 0.43, 95% CI = 0.25–0.73, *P* = 0.002), which remained in multivariable analysis (HR = 0.48, 95% CI: 0.28–0.84, *P* = 0.01).

## 4 Discussion

The Swedish randomised trial (SweBCG91‐RT) clearly showed that whole‐breast RT after breast‐conserving surgery decreased the risk of IBTR as compared to surgery alone [8]. In the present study, we found, in agreement with our hypothesis, that patients with tumours with low expression of HGF_cyt_ or pMet_cyt_ derived a substantially higher benefit from RT compared to patients with high expression of these proteins. This was most evident when restricting the follow‐up to the first 5 years. However, in contrast with our hypothesis, patients with tumours expressing high levels of pAkt_nuc_ experienced a larger treatment benefit than those with low expression and the analysis after 10 years of follow‐up suggested an interaction between pAkt_nuc_ and treatment (*P* = 0.013). When considering any recurrence as endpoint, the pattern was similar but less pronounced.

Based on the biomarkers investigated, the risk of IBTR following RT in the subgroups with less marked treatment benefit was of a magnitude that might motivate intensified treatment, maybe in conjunction with other risk factors of IBTR, such as young age and high histological grade. Given the low use of systemic adjuvant treatment in SweBCG91‐RT, intensified treatment should be in the form of current systemic adjuvant treatment, possibly in combination with an RT boost, which has been shown to decrease the risk of IBTR after breast‐conserving therapy and whole‐breast RT [23]. Another option could be to increase the radiosensitivity of the tumour cells by adding a treatment targeting a protein that contributes to radioresistance. Experimental studies have indicated that radiation can induce overexpression of Met in tumour cells from several cancer forms, including breast cancer, pancreas cancer and nasopharyngeal cancer, leading to increased sensitivity to HGF and higher invasiveness [3, 4, 24]. Consistently, treatment with Met inhibitors enhanced the efficacy of radiation and prevented radiation‐induced invasiveness [3]. Similarly, in nonsmall‐cell lung cancer, responders to 2 months of RT had higher levels of microRNA‐198 in the tumour than nonresponders, and HGF/Met signalling was suggested to be a crucial mediator of this effect [25]. Nevertheless, a mechanism that might link Met to radioresistance is the enhanced DNA repair induced by HGF after radiation [26]. The potential molecular crosstalk between Met and the DNA damage response has been further reviewed by Medova *et al*. [27]. Besides these several preclinical results, indicating a relationship between Met activation and radioresistance, the inhibitor crizotinib, in one study, failed to enhance the effect of radiation in head and neck squamous cell carcinoma xenografts [28], and clinical trials combining RT with Met inhibitors are so far lacking. Our results give additional support for testing whether this approach could be beneficial in patient subgroups with tumours overexpressing the HGF/Met axis.

The activation of Met is frequently followed by the downstream activation of Akt. Moreover, Akt is activated in response to ionising radiation and promotes cell survival [29, 30]. The expression of constitutively active Akt in breast cancer cells was shown to increase cellular resistance to radiation [31] and to decrease cell death by apoptosis after radiation [32]. A direct link between Akt activation, repair of DNA damage and radioresistance has been suggested in glioblastoma [33]. There has also been some support for pAkt to predict low efficacy of RT assessed in tumour samples from patients with head and neck cancer [34] and breast cancer [35]. However, data from clinical trials concerning the link between Akt and radioresistance are limited. Since pAkt is mostly considered to be related to radioresistance, the present result that high pAkt_nuc_ predicted more benefit from RT is challenging. The same was not seen for cytoplasmic pAkt. In recent years, the picture of the interplay between Akt activation and DNA damage response and repair has become more complex [36]. In contrast to the findings described above, Akt activation was shown to suppress DNA repair via downregulation of MRE11 [37] and homologous recombination was inhibited by Akt through inducing cytoplasmic translocation of BRCA1 and RAD51 [38]. Furthermore, nonhomologous end‐joining DNA repair might be impaired by Akt‐mediated phosphorylation of XLF [39]. Interestingly, different forms of activating *AKT1* mutants were shown to have opposite effects on DNA double‐strand break repair and radiosensitivity [12].

Our results, together with previous results [12], suggest that the cellular localisation of pAkt could be of importance for radiosensitivity. In the context of breast cancer, it is also relevant to consider the crosstalk linking the DNA damage response and repair machinery and oestrogen signalling pathways [40]. Steroid hormones can both positively and negatively regulate homologous recombination and to positively regulate nonhomologous end‐joining. ATR is functionally downregulated and CHK1 phosphorylated by ER transactivated Akt signalling, which suppresses DNA damage‐induced actions [41]. Oestrogen together with Akt signalling thus may increase the radiosensitivity by overriding cell cycle checkpoints.

Although high HGF_str_, high pMet_mem_ and high pAkt_cyt_ were associated with more aggressive tumour characteristics, these markers were not associated with poor prognosis (both endpoints) in the group of patients not treated with RT. For HGF_cyt_, the trend was rather the opposite for the endpoints analysed. In other studies of breast cancer, high expression of HGF has been associated with either poor [42] or favourable prognosis [43, 44] and high levels of HGF in serum were associated with longer relapse‐free survival after neoadjuvant chemotherapy [45]. Considering the different endpoints, the prognosis for patients in the control group was not associated with levels of pMet. For total Met expression, it was concluded from a meta‐analysis that Met overexpression is an adverse prognostic marker in breast cancer with the strongest association for triple‐negative disease [43]. Likewise, in a previous study, we found that gene copy gain of *MET* was associated with adverse prognosis, especially for patients treated with adjuvant chemotherapy [17]. It was also found that gene copy gain of both *MET* and *HGF* predicted more benefit from RT *vs*. chemotherapy than those without copy gain regarding locoregional recurrence. In contrast to the present study, the patients were all treated with mastectomy and the vast majority had lymph node‐positive disease. It is not clear from the meta‐analysis to what extent the patients in the different studies received adjuvant therapy [43]. In our study, the majority (92%) of the patients did not receive adjuvant systemic therapy.

Potential limitations of this study include that the majority of the patients did not receive adjuvant systemic therapy, which is known to decrease the risk of recurrence further. The lack of systemic therapy makes the absolute rates of recurrences presented herein difficult to interpret in a modern setting, where the majority of the patient included in SweBCG91‐RT would have been treated with adjuvant systemic therapy. However, this cohort is uniquely suited to address the question of radioresistance without the confounding of other types of treatment. As such, we believe that this study provides valuable information of radioresistance mediated by HGF, pMet and pAkt in patient tumour samples, but clearly, further studies are needed to determine how this could be implemented in clinical practice. The high number of statistical analyses also needs to be considered when interpreting the results, as this increases the risk for false‐positive findings, and the results need to be confirmed in future studies.

## 5 Conclusions

In conclusion, low expression of HGF or pMet may indicate a larger benefit from RT as compared with high expression of the proteins. The same may be true for a high level of pAkt in the nucleus. A subgroup of patients with no benefit from RT could not be identified in this study. Thus, the biomarkers might be more useful for identifying patients for intensified therapy rather than for de‐escalation purposes, and these biomarkers represent targetable proteins with already existing inhibitors that could potentially be used in conjunction with radiotherapy.

## Conflict of interest

The authors declare no conflict of interest.

## Author contributions

MS, CV, MF, PM and OS conceived and designed the study. PM, FK and PK performed clinical data assembly. CV and OS performed the expression scoring. MS and EH performed the statistical analysis. All authors interpreted the results, critically revised and approved the final version of the manuscript.

### Peer Review

The peer review history for this article is available at https://publons.com/publon/10.1002/1878‐0261.12803.

## Supporting information


**Fig. S1.** Prognostic effect of different levels of HGF_str_ (A, B), HGF_cyt_ (C, D), pMet_cyt_ (E, F), pMet_mem_ (G, H), pAkt_cyt_ (I, J), and pAkt_nuc_ (K, L) for IBTR in patients treated with or without adjuvant whole‐breast radiotherapy (RT) in the SweBCG91‐RT study.
**Fig. S2.** Prognostic effect of different levels of HGF_str_ (A, B), HGF_cyt_ (C, D), pMet_cyt_ (E, F), pMet_mem_ (G, H), pAkt_cyt_ (I, J), and pAkt_nuc_ (K, L) for any recurrence in patients treated with or without adjuvant whole‐breast radiotherapy (RT) in the SweBCG91‐RT study.

## References

[1] Early Breast Cancer Trialists' Collaborative Group , Darby S , McGale P , Correa C , Taylor C , Arriagada R , Clarke M , Cutter D , Davies C , Ewertz M *et al* (2011) Effect of radiotherapy after breast‐conserving surgery on 10‐year recurrence and 15‐year breast cancer death: meta‐analysis of individual patient data for 10,801 women in 17 randomised trials. Lancet 378, 1707–1716. 22019144 10.1016/S0140-6736(11)61629-2 PMC3254252

[2] Forker LJ , Choudhury A & Kiltie AE (2015) Biomarkers of tumour radiosensitivity and predicting benefit from radiotherapy. Clin Oncol (R Coll Radiol) 27, 561–569. 26119726 10.1016/j.clon.2015.06.002

[3] De Bacco F , Luraghi P , Medico E , Reato G , Girolami F , Perera T , Gabriele P , Comoglio PM & Boccaccio C (2011) Induction of MET by ionizing radiation and its role in radioresistance and invasive growth of cancer. J Natl Cancer Inst 103, 645–661. 21464397 10.1093/jnci/djr093

[4] Liu T , Li Q , Sun Q , Zhang Y , Yang H , Wang R , Chen L & Wang W (2014) MET inhibitor PHA‐665752 suppresses the hepatocyte growth factor‐induced cell proliferation and radioresistance in nasopharyngeal carcinoma cells. Biochem Biophys Res Commun 449, 49–54. 24802404 10.1016/j.bbrc.2014.04.147

[5] Saigusa S , Toiyama Y , Tanaka K , Yokoe T , Fujikawa H , Matsushita K , Okugawa Y , Inoue Y , Uchida K , Mohri Y *et al* (2012) Inhibition of HGF/cMET expression prevents distant recurrence of rectal cancer after preoperative chemoradiotherapy. Int J Oncol 40, 583–591. 21922134 10.3892/ijo.2011.1200

[6] Bussink J , van der Kogel AJ & Kaanders JH (2008) Activation of the PI3‐K/AKT pathway and implications for radioresistance mechanisms in head and neck cancer. Lancet Oncol 9, 288–296. 18308254 10.1016/S1470-2045(08)70073-1

[7] Toulany M & Rodemann HP (2015) Phosphatidylinositol 3‐kinase/Akt signaling as a key mediator of tumor cell responsiveness to radiation. Semin Cancer Biol 35, 180–190. 26192967 10.1016/j.semcancer.2015.07.003

[8] Malmstrom P , Holmberg L , Anderson H , Mattsson J , Jonsson PE , Tennvall‐Nittby L , Balldin G , Loven L , Svensson JH , Ingvar C *et al* (2003) Breast conservation surgery, with and without radiotherapy, in women with lymph node‐negative breast cancer: a randomised clinical trial in a population with access to public mammography screening. Eur J Cancer 39, 1690–1697. 12888363 10.1016/s0959-8049(03)00324-1

[9] Killander F , Karlsson P , Anderson H , Mattsson J , Holmberg E , Lundstedt D , Holmberg L & Malmstrom P (2016) No breast cancer subgroup can be spared postoperative radiotherapy after breast‐conserving surgery. Fifteen‐year results from the Swedish Breast Cancer Group randomised trial, SweBCG 91 RT. Eur J Cancer 67, 57–65. 27614164 10.1016/j.ejca.2016.08.001

[10] Sjostrom M , Lundstedt D , Hartman L , Holmberg E , Killander F , Kovacs A , Malmstrom P , Nimeus E , Werner Ronnerman E , Ferno M *et al* (2017) Response to radiotherapy after breast‐conserving surgery in different breast cancer subtypes in the Swedish breast cancer group 91 radiotherapy randomized clinical trial. J Clin Oncol 35, 3222–3229. 28759347 10.1200/JCO.2017.72.7263

[11] Sjostrom M , Chang SL , Fishbane N , Davicioni E , Zhao SG , Hartman L , Holmberg E , Feng FY , Speers CW , Pierce LJ *et al* (2019) Clinicogenomic radiotherapy classifier predicting the need for intensified locoregional treatment after breast‐conserving surgery for early‐stage breast cancer. J Clin Oncol 37, 3340–3349. 31618132 10.1200/JCO.19.00761 PMC6901281

[12] Oeck S , Al‐Refae K , Riffkin H , Wiel G , Handrick R , Klein D , Iliakis G & Jendrossek V (2017) Activating Akt1 mutations alter DNA double strand break repair and radiosensitivity. Sci Rep 7, 42700. 28209968 10.1038/srep42700 PMC5314324

[13] Bostner J , Karlsson E , Pandiyan MJ , Westman H , Skoog L , Fornander T , Nordenskjöld B & Stål O (2013) Activation of Akt, mTOR, and the estrogen receptor as a signature to predict tamoxifen treatment benefit. Breast Cancer Res Treat 137, 397–406. 23242584 10.1007/s10549-012-2376-y PMC3539073

[14] Ginty F , Adak S , Can A , Gerdes M , Larsen M , Cline H , Filkins R , Pang Z , Li Q & Montalto MC (2008) The relative distribution of membranous and cytoplasmic met is a prognostic indicator in stage I and II colon cancer. Clin Cancer Res 14, 3814–3822. 18559601 10.1158/1078-0432.CCR-08-0180

[15] Elston CW & Ellis IO (1991) Pathological prognostic factors in breast cancer. I. The value of histological grade in breast cancer: experience from a large study with long‐term follow‐up. Histopathology 19, 403–410. 1757079 10.1111/j.1365-2559.1991.tb00229.x

[16] McShane LM , Altman DG , Sauerbrei W , Taube SE , Gion M , Clark GM & Statistics Subcommittee of the NCI‐EORTC Working Group on Cancer Diagnostics (2005) Reporting recommendations for tumor marker prognostic studies (REMARK). J Natl Cancer Inst 97, 1180–1184. 16106022 10.1093/jnci/dji237

[17] Veenstra C , Perez‐Tenorio G , Stelling A , Karlsson E , Mirwani SM , Nordenskoljd B , Fornander T & Stal O (2016) Met and its ligand HGF are associated with clinical outcome in breast cancer. Oncotarget 7, 37145–37159. 27175600 10.18632/oncotarget.9268 PMC5095065

[18] R Core Team (2019) R: A Language and Environment for Statistical Computing. R Core Team, Vienna.

[19] Gray B (2019) cmprsk: Subdistribution analysis of competing risks. R package version 2.2‐9. Available at: http://CRAN.R‐project.org/package=cmprsk

[20] Therneau T (2015) A package for survival analysis in S. Version 2.38. http://CRANR‐projectorg/package=survival

[21] Sjostrom M , Chang SL , Fishbane N , Davicioni E , Hartman L , Holmberg E , Feng FY , Speers CW , Pierce LJ , Malmstrom P *et al* (2020) Comprehensive transcriptomic profiling identifies breast cancer patients who may be spared adjuvant systemic therapy. Clin Cancer Res 26, 171–182. 31558478 10.1158/1078-0432.CCR-19-1038

[22] Gordon M & Lumley T (2019) forestplot: Advanced forest plot using ‘grid’ graphics. R package version 1.9.

[23] Bartelink H , Maingon P , Poortmans P , Weltens C , Fourquet A , Jager J , Schinagl D , Oei B , Rodenhuis C , Horiot JC *et al* (2015) Whole‐breast irradiation with or without a boost for patients treated with breast‐conserving surgery for early breast cancer: 20‐year follow‐up of a randomised phase 3 trial. Lancet Oncol 16, 47–56. 25500422 10.1016/S1470-2045(14)71156-8

[24] Qian LW , Mizumoto K , Inadome N , Nagai E , Sato N , Matsumoto K , Nakamura T & Tanaka M (2003) Radiation stimulates HGF receptor/c‐Met expression that leads to amplifying cellular response to HGF stimulation via upregulated receptor tyrosine phosphorylation and MAP kinase activity in pancreatic cancer cells. Int J Cancer 104, 542–549. 12594808 10.1002/ijc.10997

[25] Zhu YC , Wang WX , Xu CW , Zhuang W , Song ZB , Du KQ , Chen G , Lv TF & Song Y (2018) A novel co‐existing ZCCHC8‐ROS1 and de‐novo MET amplification dual driver in advanced lung adenocarcinoma with a good response to crizotinib. Cancer Biol Ther 19, 1097–1101. 30095326 10.1080/15384047.2018.1491506 PMC6301800

[26] Fan S , Ma YX , Wang JA , Yuan RQ , Meng Q , Cao Y , Laterra JJ , Goldberg ID & Rosen EM (2000) The cytokine hepatocyte growth factor/scatter factor inhibits apoptosis and enhances DNA repair by a common mechanism involving signaling through phosphatidyl inositol 3' kinase. Oncogene 19, 2212–2223. 10822371 10.1038/sj.onc.1203566

[27] Medova M , Aebersold DM & Zimmer Y (2013) The molecular crosstalk between the MET receptor tyrosine kinase and the DNA damage response‐biological and clinical aspects. Cancers (Basel) 6, 1–27. 24378750 10.3390/cancers6010001 PMC3980615

[28] Baschnagel AM , Galoforo S , Thibodeau BJ , Ahmed S , Nirmal S , Akervall J & Wilson GD (2015) Crizotinib fails to enhance the effect of radiation in head and neck squamous cell carcinoma xenografts. Anticancer Res 35, 5973–5982. 26504020

[29] Valerie K , Yacoub A , Hagan MP , Curiel DT , Fisher PB , Grant S & Dent P (2007) Radiation‐induced cell signaling: inside‐out and outside‐in. Mol Cancer Ther 6, 789–801. 17363476 10.1158/1535-7163.MCT-06-0596

[30] Bozulic L , Surucu B , Hynx D & Hemmings BA (2008) PKBalpha/Akt1 acts downstream of DNA‐PK in the DNA double‐strand break response and promotes survival. Mol Cell 30, 203–213. 18439899 10.1016/j.molcel.2008.02.024

[31] Liang K , Jin W , Knuefermann C , Schmidt M , Mills GB , Ang KK , Milas L & Fan Z (2003) Targeting the phosphatidylinositol 3‐kinase/Akt pathway for enhancing breast cancer cells to radiotherapy. Mol Cancer Ther 2, 353–360. 12700279

[32] Soderlund K , Perez‐Tenorio G & Stal O (2005) Activation of the phosphatidylinositol 3‐kinase/Akt pathway prevents radiation‐induced apoptosis in breast cancer cells. Int J Oncol 26, 25–32. 15586221

[33] Kao GD , Jiang Z , Fernandes AM , Gupta AK & Maity A (2007) Inhibition of phosphatidylinositol‐3‐OH kinase/Akt signaling impairs DNA repair in glioblastoma cells following ionizing radiation. J Biol Chem 282, 21206–21212. 17513297 10.1074/jbc.M703042200 PMC3614065

[34] Gupta AK , McKenna WG , Weber CN , Feldman MD , Goldsmith JD , Mick R , Machtay M , Rosenthal DI , Bakanauskas VJ , Cerniglia GJ *et al* (2002) Local recurrence in head and neck cancer: relationship to radiation resistance and signal transduction. Clin Cancer Res 8, 885–892. 11895923

[35] Stal O , Perez‐Tenorio G , Akerberg L , Olsson B , Nordenskjold B , Skoog L & Rutqvist LE (2003) Akt kinases in breast cancer and the results of adjuvant therapy. Breast Cancer Res 5, R37–R44. 12631397 10.1186/bcr569 PMC154147

[36] Szymonowicz K , Oeck S , Malewicz NM & Jendrossek V (2018) New insights into protein kinase B/Akt signaling: role of localized Akt activation and compartment‐specific target proteins for the cellular radiation response. Cancers (Basel) 10, 78. 10.3390/cancers10030078 PMC5876653 29562639

[37] Piscitello D , Varshney D , Lilla S , Vizioli MG , Reid C , Gorbunova V , Seluanov A , Gillespie DA & Adams PD (2018) AKT overactivation can suppress DNA repair via p70S6 kinase‐dependent downregulation of MRE11. Oncogene 37, 427–438. 28967905 10.1038/onc.2017.340 PMC5799716

[38] Plo I , Laulier C , Gauthier L , Lebrun F , Calvo F & Lopez BS (2008) AKT1 inhibits homologous recombination by inducing cytoplasmic retention of BRCA1 and RAD51. Cancer Res 68, 9404–9412. 19010915 10.1158/0008-5472.CAN-08-0861

[39] Liu P , Gan W , Guo C , Xie A , Gao D , Guo J , Zhang J , Willis N , Su A , Asara JM *et al* (2015) Akt‐mediated phosphorylation of XLF impairs non‐homologous end‐joining DNA repair. Mol Cell 57, 648–661. 25661488 10.1016/j.molcel.2015.01.005 PMC4336609

[40] Schiewer MJ & Knudsen KE (2016) Linking DNA damage and hormone signaling pathways in cancer. Trends Endocrinol Metab 27, 216–225. 26944914 10.1016/j.tem.2016.02.004 PMC4808434

[41] Pedram A , Razandi M , Evinger AJ , Lee E & Levin ER (2009) Estrogen inhibits ATR signaling to cell cycle checkpoints and DNA repair. Mol Biol Cell 20, 3374–3389. 19477925 10.1091/mbc.E09-01-0085 PMC2710824

[42] Yamashita J , Ogawa M , Yamashita S , Nomura K , Kuramoto M , Saishoji T & Shin S (1994) Immunoreactive hepatocyte growth factor is a strong and independent predictor of recurrence and survival in human breast cancer. Cancer Res 54, 1630–1633. 8137271

[43] Yan S , Jiao X , Zou H & Li K (2015) Prognostic significance of c‐Met in breast cancer: a meta‐analysis of 6010 cases. Diagn Pathol 10, 62. 26047809 10.1186/s13000-015-0296-y PMC4458003

[44] Lengyel E , Prechtel D , Resau JH , Gauger K , Welk A , Lindemann K , Salanti G , Richter T , Knudsen B , Vande Woude GF *et al* (2005) C‐Met overexpression in node‐positive breast cancer identifies patients with poor clinical outcome independent of Her2/neu. Int J Cancer 113, 678–682. 15455388 10.1002/ijc.20598

[45] Kim H , Youk J , Yang Y , Kim TY , Min A , Ham HS , Cho S , Lee KH , Keam B , Han SW *et al* (2016) Prognostic implication of serum hepatocyte growth factor in stage II/III breast cancer patients who received neoadjuvant chemotherapy. J Cancer Res Clin Oncol 142, 707–714. 26577828 10.1007/s00432-015-2072-5 PMC11819070

