# Supplementary material for: Expression of HGF, pMet, and pAkt is related to benefit of radiotherapy after breast‐conserving surgery: a long‐term follow‐up of the SweBCG91‐RT randomised trial
Source: Mol Oncol. 2020 Sep 28;14(11):2713–26. doi: 10.1002/1878-0261.12803 (PMC7607179; doi:10.1002/1878-0261.12803)
Supplement: Supplementary file 1 — Fig. S1. Prognostic effect of different levels of HGFstr (A, B), HGFcyt (C, D), pMetcyt (E, F), pMetmem (G, H), pAktcyt (I, J), and pAktnuc (K, L) for IBTR in patients treated with or without adjuvant whole‐breast radiotherapy (RT) in the SweBCG91‐RT study. Fig. S2. Prognostic effect of different levels of HGFstr (A, B), HGFcyt (C, D), pMetcyt (E, F), pMetmem (G, H), pAktcyt (I, J), and pAktnuc (K, L) for any recurrence in patients treated with or without adjuvant whole‐breast radiotherapy (RT) in the SweBCG91‐RT study. [file MOL2-14-2713-s001.pdf]

Supplementary Figure 1

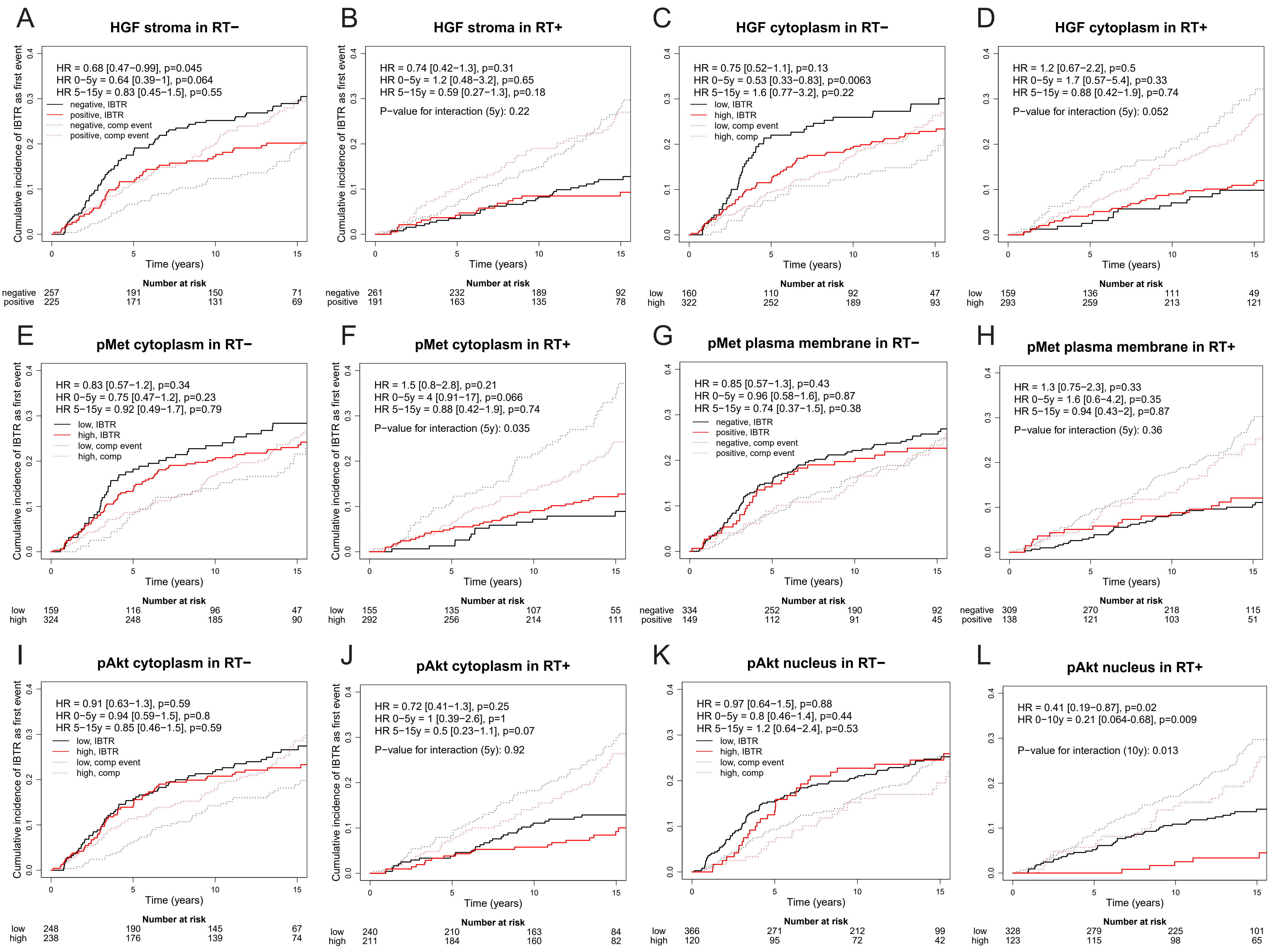

Supplementary Figure 1

Prognostic effect of different levels of HGF_{str} (A, B), HGF_{cyt} (C, D), $pMet_{cyt}$ (E, F), $pMet_{mem}$ (G, H), $pAkt_{cyt}$ (I, J), and $pAkt_{nuc}$ (K, L) for IBTR in patients treated with or without adjuvant whole-breast radiotherapy (RT) in the SweBCG91-RT study. Solid lines represent the cumulative incidence of IBTR, while dashed lines represent the cumulative incidence of competing events.

Supplementary Figure 2

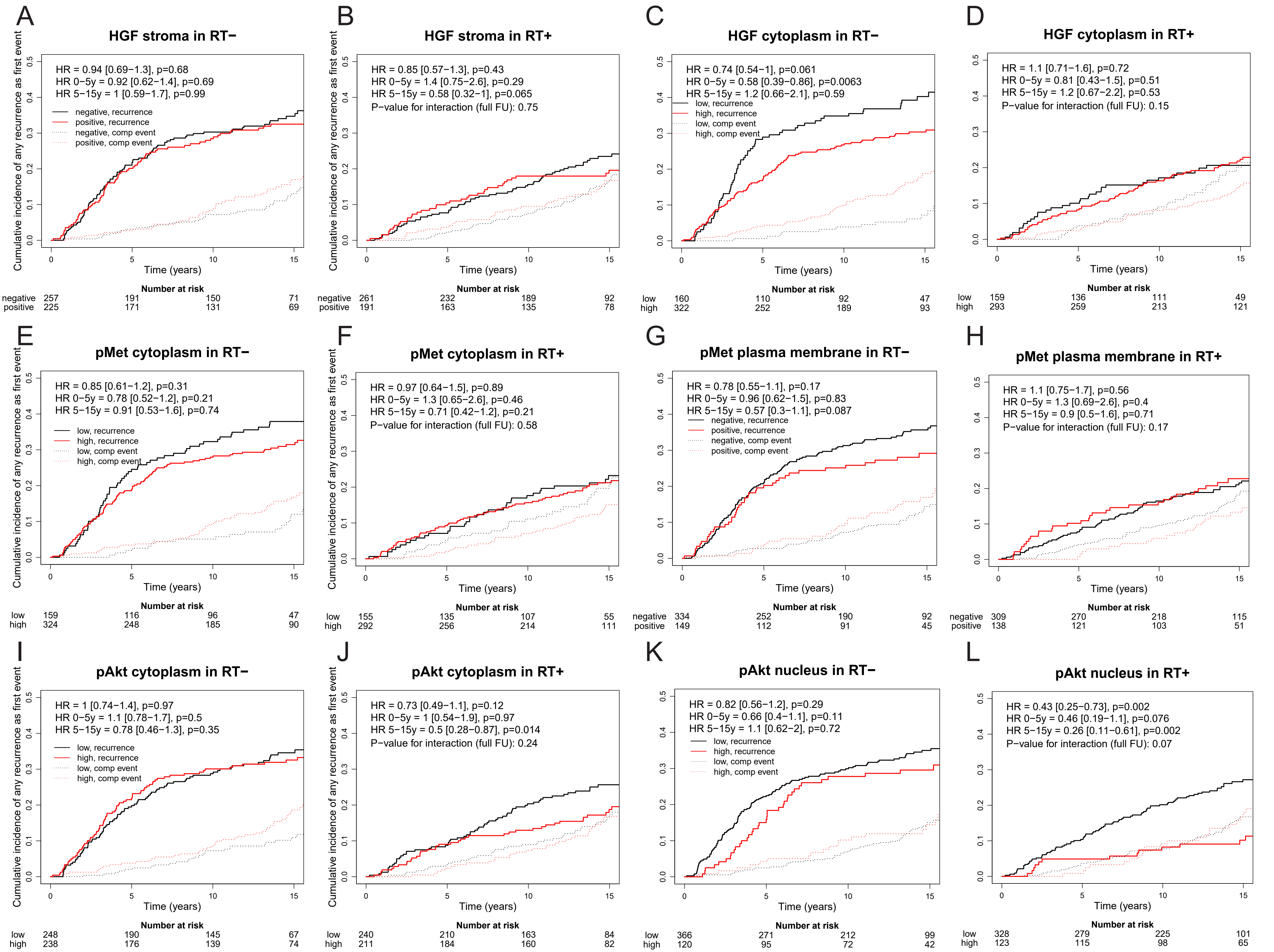

Supplementary Figure 2

Prognostic effect of different levels of HGF_{str} (A, B), HGF_{cyt} (C, D), $pMet_{cyt}$ (E, F), $pMet_{mem}$ (G, H), $pAkt_{cyt}$ (I, J), and $pAkt_{nuc}$ (K, L) for any recurrence in patients treated with or without adjuvant whole-breast radiotherapy (RT) in the SweBCG91-RT study. Solid lines represent the cumulative incidence of IBTR, while dashed lines represent the cumulative incidence of competing events.
